# OrganelX web server for sub-peroxisomal and sub-mitochondrial protein localization and peroxisomal target signal detection

**DOI:** 10.1016/j.csbj.2022.11.058

**Published:** 2022-12-05

**Authors:** Marco Anteghini, Asmaa Haja, Vitor A.P. Martins dos Santos, Lambert Schomaker, Edoardo Saccenti

**Affiliations:** a Laboratory of Systems and Synthetic Biology, Wageningen University & Research, Wageningen, The Netherlands; b LifeGlimmer GmbH, Berlin, Germany; c Bernoulli Institute, University of Groningen, Groningen, The Netherlands; d Bioprocess Engineering, Wageningen University & Research, Wageningen, The Netherlands

**Keywords:** Sub-cellular localization, Sub-peroxisomal localization, Sub-mithocondrial localization, Peroxisomal-targeting-signal, Peroxisome

## Abstract

We present the OrganelX e-Science Web Server that provides a user-friendly implementation of the In-Pero and In-Mito classifiers for sub-peroxisomal and sub-mitochondrial localization of peroxisomal and mitochondrial proteins and the Is-PTS1 algorithm for detecting and validating potential peroxisomal proteins carrying a PTS1 signal sequence. The OrganelX e-Science Web Server is available at https://organelx.hpc.rug.nl/fasta/.

## 1 Introduction

Signatures in the amino acid sequences of proteins have been associated with domains, family functional sites and their sub-cellular localization [1], [2], [3], [4]. These sequences can be used in association with machine learning (ML) approaches to develop prediction tools, that nowadays are easily findable and accessible [5], [6], [7], [8]. Deep-learning approaches have been recently used to embed (encode) the protein sequences, which showed promising results for several tasks, including sub-cellular classification [9], [10], [11], [12], [13], [14], [15]. The Unified Representation (UniRep) [9] and the Sequence-to-Vector (SeqVec) [10] are two of the most promising and already used protein sequence embeddings. UniRep provides an amino-acid embedding that summarizes physico-chemical properties and phylogenetic clusters and has been shown to be efficient for distinguishing proteins from various structural classifications of protein classes [9]. SeqVec showed optimal performance for predicting sub-cellular localisation [10]. The potential of these embeddings has been recently explored for highly specific tasks, such as sub-organelle localisation: in particular, they have been used for sub-peroxisomal and sub-mitochondrial protein localisation [16]. Peroxisomes and mitochondria are ubiquitous organelles surrounded by a single (peroxisomes) or a double (mitochondria) biomembrane that is relevant to many metabolic and non-metabolic pathways [17], [18]. The full extent of the functions of peroxisomes, mitochondria and of the involved pathways is still largely unknown [19]: in this light, the discovery of new peroxisomal and mitochondrial proteins can facilitate further knowledge acquisition. Here we present the OrganelX Web Server (available at https://organelx.hpc.rug.nl/fasta/) which hosts two existing algorithms designed to predict sub-peroxisomal (In-Pero) and sub-mitochondrial (In-Mito) localization of a (set of) protein(s) starting from the amino acid sequence(s). The In-Pero and In-Mito algorithm have been introduced in [16] and can be used to predict the sub-cellular localization of known or putative peroxisomal and mitochondrial proteins whose localization is unknown. We also introduce a new functionality (the Is-PTS1 algorithm) for the classification of protein sequences as peroxisomal (*i.e.* proteins that can be imported in the peroxisome) or non-peroxisomal starting from the detection of a specific peroxisomal targeting signal (PTS1) [20].

To our knowledge, there are no online resources that allow simple and fast prediction of the sub-peroxisomal and sub-mitochondrial localization or the prediction of peroxisomal proteins through identification of the PST1 signal starting from the amino acid sequence. These tools offered online through the OrganelX server facilitate research on peroxisomes and mitochondria by making the prediction of protein sub-cellular localisation easy to perform (only the upload of protein FASTA sequences is needed). OrganelX can be used without the need for programming skills and significantly reduces the number of bioinformatic steps that should have been otherwise performed to extract relevant information from the protein sequences of interest [21].

## 2 Materials and Methods

### 2.1 In-Pero and In-Mito classifiers

The In-Pero and In-Mito algorithms and the prediction models implemented in the OrganelX Web Server have been introduced and described in Anteghini et al. (2021) [16]. We give here a brief account of the most important characteristics. We refer the reader to the original publication for full details on algorithm development, training and validation.

The In-Pero prediction model was originally trained on a curated, non-redundant (40% of sequence identity) data set of 160 peroxisomal proteins [16] with validated sub-cellular localization; the In-Mito model was trained on a curated, non-redundant (40% of sequence identity) data set of 424 mitochondrial proteins [16] also with validated sub-cellular localization.

Both algorithms start by encoding the protein amino acid sequence using the concatenation of two deep learning-based sequence embeddings [16]: UniRep [9] and Seqvec [10] [9], [10], [16].

The classification problems are solved using Support Vector Machines [22]. The In-Pero algorithm predicts whether a proximal protein belongs to the matrix or is a (trans) membrane protein, resulting in a binary classification problem. The In-Mito algorithm predicts the possible localization of mitochondrial proteins: matrix, inner-membrane, inter-membrane and outer-membrane, resulting in a four-class classification problem.

### 2.2 The Is-PTS1 classifier

The proteins that are imported into peroxisomes (peroxisomal proteins) are directed to the peroxisome through the PEX5 receptor that recognizes a specific region of the peroxisomal protein called a peroxisomal targeting signal 1 (PTS1) [23]. Operationally, the PTS1 is defined as dodecamer sequences at the C-terminal ends of the protein sequence which accommodate physical contacts with both the surface and the binding cavity of PEX5 and ensure accessibility of the extreme C-terminus [20]. However, the presence of the PTS1 is not a guarantee for the import of proteins across the peroxisomal membrane [24], [25], [20]. The problem is then to predict whether a protein carrying the PTS1 is a peroxisomal protein or not.

The Is-PTS1 algorithm first searches putative PTS1 signals by matching a regular expression [ASCNPHTGEQ][RKHQNSL][LAMIVF] in the C-terminal part of the sequence (last 3 amino acids) [21], [26].

Due to some limitations in the embedding generation procedure, we recommend the user upload sequences with less than 1200 residues [9], [10]. When dealing with longer sequences we recommend conserving the C-terminal part and eventually removing the N-terminal part.

If the PTS1 signal is found, the full amino acid sequence is encoded using the concatenation of UniRep [9] and Seqvec [10] protein embeddings [9], [10] as in the case of the In-Pero and In-Mito algorithms [16]. The binary classification of the protein sequence as peroxisomal or not-peroxisomal is carried over using a Support Vector Machine classifier [22] trained on a non-redundant (40% of sequence identity) data set consisting of 72 peroxisomal proteins (positives) and 155 non-peroxisomal proteins (negatives) all carrying a putative PTS1 signal.

An additional data set of 5 different proteomes of five organisms (*saccharomyces cerevisiae*, *homo sapiens*, *danio rerio*, *mus musculus* and *bos taurus*) was assembled to assess how many proteins contain a putative PTS1 signal. The protein sequence was downloaded from UniProt [27] (release 04_2022) and only the reviewed sequences were considered. An overview of the number of proteins containing a PTS1 signal is reported in Table 1. Considering the proteins from all the species, 6.4% of the reviewed protein carrying a putative PTS1 signal are also annotated as peroxisomal.

**Table 1:** Summary statistics (per organism) of proteins with the putative PTS1 signals retrieved from UniProt. ‘n. protein’ indicates the total number of proteins retrieved per organism, the peroxisomal proteins are in brackets; ‘n. matches’ the number of proteins containing a putative PTS1 signal in the C-terminal part of the sequence; ‘true matches’ (TM) indicates how many among the ‘n. of matches’ are annotated as peroxisomal.

**Organism**	**n. proteins (pero)**	**n. matches**	**true matches**	**% TM**
*s. cerevisiae*	6050 (85)	74	21	28
*homo sapiens*	20360 (143)	1180	59	5
*danio rerio*	3216 (22)	158	8	5
*mus musculus*	17085 (146)	976	63	6
*bos taurus*	6015 (53)	297	22	7

### 2.3 Model optimization

The training, hyper-parameters optimization and validation procedures of the In-Pero, In-Mito and Is-PTS prediction models were carried over using a repeated double cross-validation approach [28], [29] as detailed in [16].

### 2.4 Prediction results

The results of the prediction (Peroxisomal and Mitochondrial sub-cellular localization, presence of the PTS1/peroxisomal protein) are given with an associated probability. For the binary classifiers (In-Pero and Is-PTS1) the class probability is calibrated using Platt scaling [30] from the logistic regression on the SVM scores, fit by additional cross-validation on the training data. For the multi-class classifier (In-Mito), the class probability was calculated using the improved version of the coupling approach [31], [32].

### 2.5 Data sets for extra validation

We assembled two additional data sets for extra validation of the In-Pero and Is-PTS algorithms (Web server implementation). The In-Mito algorithm was already externally validated in the original publication against two existing tools: DeepMito [33] and DeepPred-SubMito [34] (see Table 3 in [16]).

For the validation of In-Pero, we queried UniProt [35] for reviewed proteins with a clear sub-peroxisomal annotation in the membrane (”SL-0203” and ”GO:0005778”) or matrix (”SL-0202” and ”GO:0005782”). The resulting sequences were then clustered for 40% of sequence identity with CD-hit [36]. Sequences overlapping with our original training set were removed, obtaining 85 membrane proteins and 59 matrix proteins.

To validate the Is-PTS1 algorithm we retrieved from UniProt (and processed in a similar way) 15 peroxisomal proteins carrying the PTS1 signal (true positives) and 15 non-peroxisomal proteins carrying the PTS1 signal (true negatives).

### 2.6 Software

The OrganelX Web Server was implemented using Django, a high-level Python web framework [37] (https://www.djangoproject.com/).

The internal services for running the classification algorithms are located on Peregrine, the high-performance computing cluster at the University of Groningen, the Netherlands. For more info see https://www.rug.nl/society-business/centre-for-information-technology/research/services/hpc/facilities/peregrine-hpc-cluster?lang = en).

## 3 Results

### 3.1 Performance and validation of the prediction algorithms

#### 3.1.1 Performance of In-Pero and In-Mito

The performance and benchmarking of the In-Pero and In-Mito algorithm are exhaustively illustrated and discussed in [16]. For convenience we give in Table 2 a summary of the validation results from [16].

**Table 2:** Performance of the In-Pero and In-Mito prediction algorithms from [16]. Results are given as mean ± standard deviation over a 5-fold Double Cross Validation. Prediction quality metrics: $F_{1}$ score ($F_{1}$, the harmonic mean of precision and recall), Accuracy (ACC), Matthews’ Correlation Coefficient (MCC) [38] and the Area Under the Curve (AUC). The performance of the In-Mito classifier are quantified using MCC for each sub-cellular mitochondrial compartment: outer membrane (O), inner membrane (I), inter-membrane space (T) and matrix (M). The In-Mito performances are benchmarked with two other methods namely DeepMito [33] and DeepPred-SubMito (DP-SM) [34].

**Method**	$F_{1}$	**ACC**	**MCC**	**AUC**
In-Pero	0.86±0.03	0.92±0.01	0.72±0.06	0.91±0.02
	**MCC (O)**	**MCC (I)**	**MCC (T)**	**MCC (M)**
DeepMito	0.46	0.47	0.53	0.65
DP-SM	0.85	0.49	0.99	0.56
In-Mito	0.64	0.69	0.62	0.80

#### 3.1.2 Validation of the Performance of the Is-PTS1 algorithm

The Is-PTS1 predictor is a newly implemented algorithm. Its overall performance was assessed against the data set containing peroxisomal protein carrying a PTS1 (see Table 1). The yeast peroxisome is the organelle with the highest protein concentrations which partially explain the high quantity of annotated peroxisomal protein carrying a PTS1 signal found in Uniprot [39]. Also, peroxisomal proteins are often studied on yeast as a model organism [40]. Is-PTS1 performance on the indicated data set is excellent: ACC=0.92±0.01 (Accuracy), $F_{1}=0.91\pm 0.01$ ($F_{1}$ score), AUC=0.92±0.02 (Area Under the Curve) and MCC=0.92±0.01 (Matthews’ Correlation Coefficient). Results are averaged over 5 cross-validation splits.

### 3.2 Extra validation of the Web server implementation

The performance of the In-Pero predictor on the extra validation data set is given in Table 3: the quality metrics are in line with what observed in the original publication [16]. The performance of the Is-PTS1 predictor is consistent with the results obtained in the training data set (see Section 3.1.2).

**Table 3:** Performances of the In-Pero and Is-PTS1 predictor on two extra validation data sets. Performance quality metrics: $F_{1}$ score ($F_{1}$), Accuracy (ACC), Matthews’ Correlation Coefficient (MCC) [38] and Area under the curve (AUC).

**Tools**	F1	**ACC**	**MCC**	**AUC**
In-Pero	0.83	0.88	0.74	0.86
Is-PTS1	0.84	0.83	0.67	0.83

### 3.3 Using the OrganelX Web Server

**Fig. 1:**
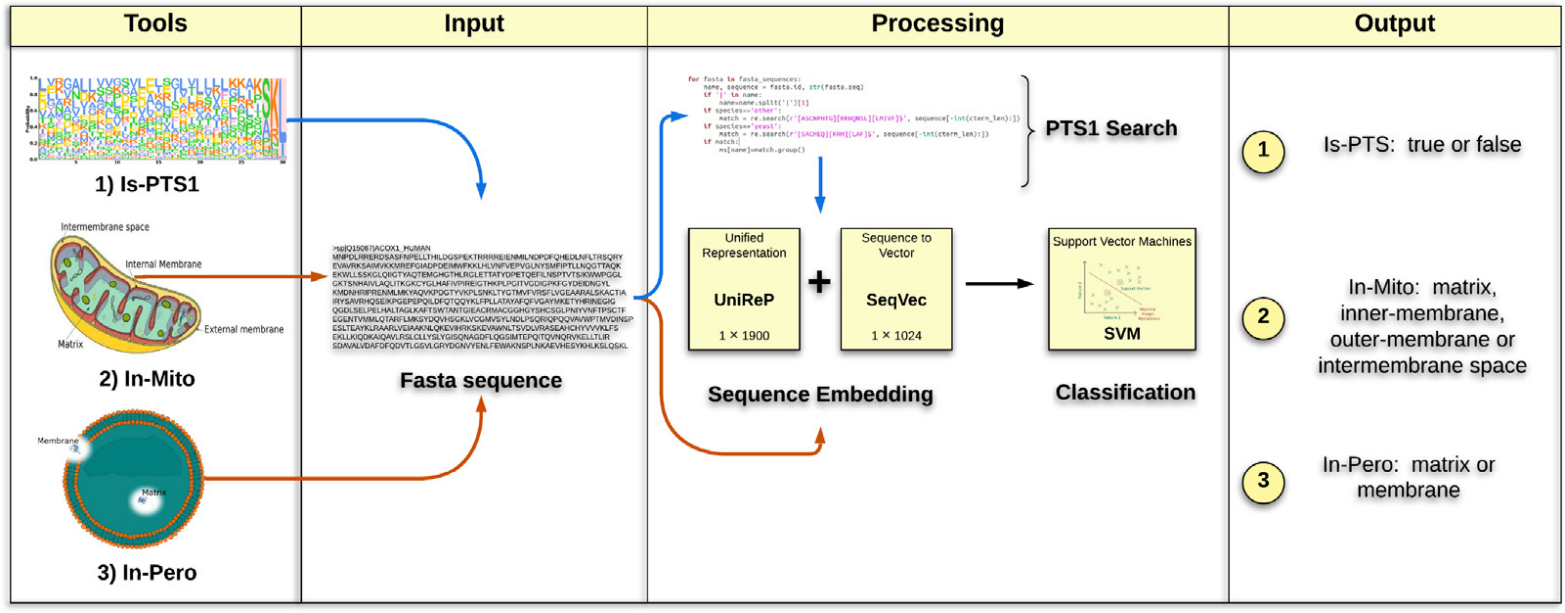
Overview of the OrganelX e-Science Web Server. The workflow of prediction of protein sub-cellular localization (In-Pero and In-Mito) and Peroxisomal Target Signal detection (Is-PTS1) is organized in four main steps: (1) Selection of the appropriate prediction algorithm; (2) Input (upload) of FASTA file containing one or more protein sequence; (3) Embedding of the protein sequence(s) and SVM-based classification; (4) Generation and presentation of the result output.

**Fig. 2:**
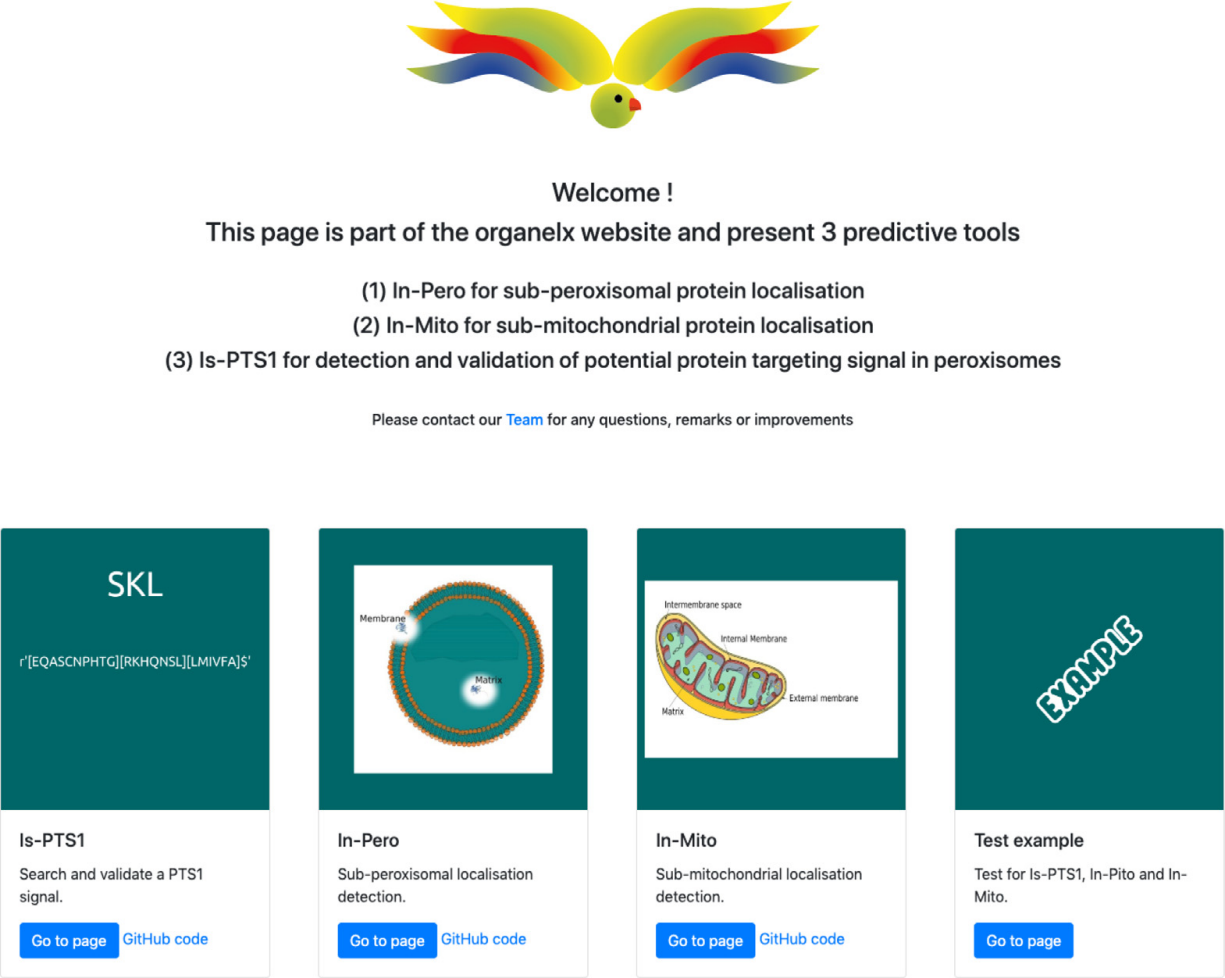
Homepage of the OrganelX Web Server (https://organelx.hpc.rug.nl/fasta/). From the homepage, the user can access the three prediction tools via the: Is-PTS1 (prediction of peroxisomal proteins based on the presence of the Peroxisome Target Signal), In-Pero (sub-cellular localization of peroxisomal proteins) and In-Mito (sub-cellular localization of peroxisomal proteins). An example is also available. Is-PTS1, In-Pero, In-Mito predictor tools as well as visualize an example. The blue buttons ‘Go to page’ redirect the user to the specific tool.

An overview of the functionalities available OrganelX Web Server is shown in Fig. 1. The different prediction tools (In-Pero, In-Mito and Is-PTS1) are accessible from the homepage as shown in Fig. 2.

#### 3.3.1 OrganelX Web Server: input

**Fig. 3:**
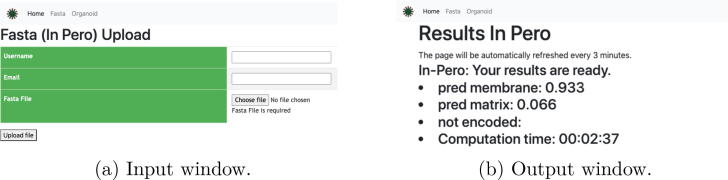
Input and output windows of the Organelx Web Server. (A) the user can specify an arbitrary username, an email address where to receive the results. The FASTA file is uploaded by clicking on the the ‘Choose file’ button; (B) the probabilities for each protein in the FASTA file will appear next to a specific class (e.g. ‘pred membrane’ or ‘pred matrix’). In case of errors during the embedding generation, the protein ID will be flagged as ‘not encoded’.

The input for the In-Pero (sub-cellular localization of peroxisomal proteins), In-Mito (sub-cellular localization of mitochondrial proteins) and Is-PTS1 (detection of a peroxisomal targeting signal) algorithms available on the OrganelX Web Server is a FASTA text file containing one or more protein sequence. Each sequence begins with a single-line description, followed by amino-acid sequence data. The single-line description consists of  > **sp—ID—Desc** symbols, where  > **sp** is a fixed prefix, **ID** is the sequence name, and **Desc** is a descriptive text, followed by tokens of the FASTA sequence on the next lines. Alternatively, the single-line description can be  > **ID** as a basic FASTA file. The input window of the OrganelX Web Server is shown in Fig. 3A.

#### 3.3.2 OrganelX Web Server: submitting a job

**Fig. 4:**
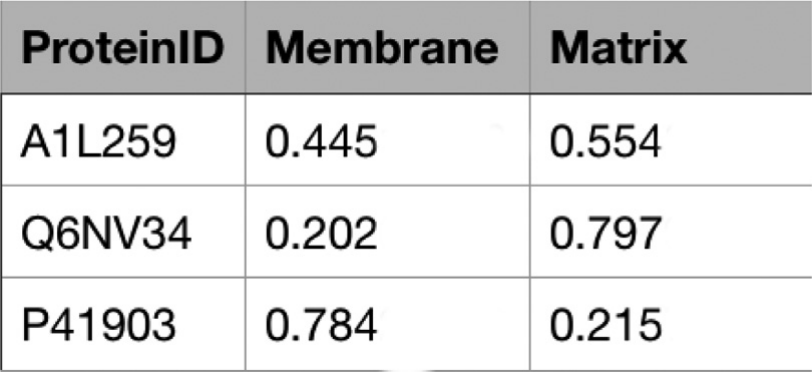
Output file in.csv format obtained from a FASTA containing 3 sequences. The column ‘ProteinID’ shows the specific UniProt ID for each entry; the columns ‘Membrane’ and ‘Matrix’ show the probability associated to the ‘Membrane’ and ‘Matrix’ classes.

When submitting a job, the user can specify a username, and an email address (optional) and upload a FASTA file. The user can either wait for the results via email or refresh the result web page. The result page is automatically refreshed every 3 min. The computation time may change depending on the file size and the traffic on the website. If an email address has been provided, the user will receive a message including the results attached in a.csv file (e.g. Fig. 4). An example can be accessed at https://organelx.hpc.rug.nl/fasta/test_example, from where an example FASTA file can be downloaded.

#### 3.3.3 OrganelX Web Server: output

The results are given in a.csv file which allows easy manipulation and re-use for further analysis. The.csv file contains the classification results and the probabilities for each predicted class. The results for each class are reported under its specific column, while each row contains the IDs of the corresponding classified entries. The output window of the OrganelX Web Server is shown in Fig. 3A.

## 4 Conclusions

The In-Pero predictor allows for accurately classifying membrane and matrix proteins inside the peroxisomes. Is-PTS1 predictor detects peroxisomal proteins carrying a PTS1 signal. The In-Mito predictor can be used as a complementary tool when investigating ambiguous or double localization in mitochondrial proteins. These tools proved to be accurate and a valid alternative to the commonly used pipelines, which are less precise, fragmented and time demanding. These three prediction algorithms are now made easily accessible and simple to use through the OrganelX Web server. OrganelX provides a solution to the problem of accurately performing sub-organelle classification and contributes to improving the lack of specific computational methods in peroxisomal research and will facilitate the work of the many groups working on peroxisome and mitochondria research.

## Availability

OrganelX e-Science Web Server can be reached at: https://organelx.hpc.rug.nl/fasta/. The data sets and stand-alone versions of the preictors (Pythone code) are available at: https://github.com/MarcoAnteghini/In-Pero(In-Pero); https://github.com/MarcoAnteghini/In-Mito(In-Mito) and https://github.com/MarcoAnteghini/Is-PTS1(Is-PTS1).

## Funding

This project has received funding from the European Union’s Horizon 2020 research and innovation program under the Marie Sklodowska-Curie grant agreement No 812968. The writing of this Chapter was partially supported by the financial contribution of The Netherlands Organization for Health Research and Development (ZonMW) under the frame of ERA PerMed (Project 2018–151, PerMIT).

## CRediT authorship contribution statement

**Marco Anteghini:** Conceptualization, Data curation, Methodology, Validation, Software, Writing - original draft, Writing - review & editing. **Asmaa Haja:** Conceptualization, Data curation, Methodology, Validation, Software, Writing - original draft. **Vitor A.P. Martins dos Santos:** Supervision, Funding acquisition. **Lambert Schomaker:** Supervision, Funding acquisition, Writing - original draft. **Edoardo Saccenti:** Conceptualization, Supervision, Writing - original draft, Writing - review & editing.

## Declaration of Competing Interest

The authors declare that they have no known competing financial interests or personal relationships that could have appeared to influence the work reported in this paper.

## References

[1] Andrade M.A., O’Donoghue S.I., Rost B. (1998). Adaptation of protein surfaces to subcellular location 1 1edited by f. e. cohen. J Mol Biol.

[2] S. Hunter, R. Apweiler, T.K. Attwood, A. Bairoch, A. Bateman, D. Binns, P. Bork, U. Das, L. Daugherty, L. Duquenne, R.D. Finn, J. Gough, D. Haft, N. Hulo, D. Kahn, E. Kelly, A. Laugraud, I. Letunic, D. Lonsdale, R. Lopez, M. Madera, J. Maslen, C. McAnulla, J. McDowall, J. Mistry, A. Mitchell, N. Mulder, D. Natale, C. Orengo, A.F. Quinn, J.D. Selengut, C.J.A. Sigrist, M. Thimma, P.D. Thomas, F. Valentin, D. Wilson, C.H. Wu, C. Yeats, InterPro: the integrative protein signature database, Nucleic Acids Research 37 (Database) (2009) D211–D215. doi:10.1093/nar/gkn785. https://doi.org/10.1093/nar/gkn785 10.1093/nar/gkn785 PMC2686546 18940856

[3] Scott M.S., Thomas D.Y., Hallett M.T. (2004). Predicting subcellular localization via protein motif co-occurrence. Genome Res.

[4] Almagro Armenteros J.J., Salvatore M., Emanuelsson O., Winther O., von Heijne G., Elofsson A., Nielsen H. (2019). Detecting sequence signals in targeting peptides using deep learning. Life Sci Alliance.

[5] Horton P., Park K.-J., Obayashi T., Fujita N., Harada H., Adams-Collier C., Nakai K. (2007). WoLF PSORT: protein localization predictor. Nucleic Acids Res.

[6] Pierleoni A., Martelli P.L., Fariselli P. (2006). Bacello: a balanced subcellular localization predictor. Bioinform (Oxford, England).

[7] Savojardo C., Martelli P.L., Fariselli P., Casadio R. (2015). TPpred3 detects and discriminates mitochondrial and chloroplastic targeting peptides in eukaryotic proteins. Bioinformatics.

[8] Y. Jiang, D. Wang, Y. Yao, H. Eubel, P. Künzler, I. Møller, D. Xu, Mulocdeep: A deep-learning framework for protein subcellular and suborganellar localization prediction with residue-level interpretation (2020). 10.1016/j.csbj.2021.08.027 PMC8426535 34522290

[9] Alley E., Khimulya G., Biswas S., Alquraishi M., Church G. (2019). Unified rational protein engineering with sequence-based deep representation learning. Nat Methods.

[10] Heinzinger M., Elnaggar A., Wang Y., Dallago C., Nechaev D., Matthes F., Rost B. (2019). Modeling aspects of the language of life through transfer-learning protein sequences. BMC Bioinform.

[11] A. Elnaggar, M. Heinzinger, C. Dallago, G. Rehawi, Y. Wang, L. Jones, T. Gibbs, T. Feher, C. Angerer, M. Steinegger, D. BHOWMIK, B. Rost, Prottrans: Towards cracking the language of life’s code through self-supervised deep learning and high performance computing, bioRxiv (2020). 10.1109/TPAMI.2021.3095381 34232869

[12] A. Rives, J. Meier, T. Sercu, S. Goyal, Z. Lin, J. Liu, D. Guo, M. Ott, C.L. Zitnick, J. Ma, R. Fergus, Biological structure and function emerge from scaling unsupervised learning to 250 million protein sequences, Proceedings of the National Academy of Sciences 118 (15) (2021) e2016239118. doi:10.1073/pnas.2016239118. doi: 10.1073/pnas.2016239118. 10.1073/pnas.2016239118 PMC8053943 33876751

[13] Savojardo C., Bruciaferri N., Tartari G., Martelli P.L., Casadio R. (2019). DeepMito: accurate prediction of protein sub-mitochondrial localization using convolutional neural networks. Bioinformatics.

[14] Almagro Armenteros J.J., Sønderby C.K., Sønderby S.K., Nielsen H., Winther O. (2017). DeepLoc: prediction of protein subcellular localization using deep learning. Bioinformatics.

[15] L. Ho Thanh Lam, N.H. Le, L. Van Tuan, H. Tran Ban, T. Nguyen Khanh Hung, N.T.K. Nguyen, L. Huu Dang, N.Q.K. Le, Machine learning model for identifying antioxidant proteins using features calculated from primary sequences, Biology 9 (10) (2020). 10.3390/biology9100325 PMC7599600 33036150

[16] Anteghini M., dos Santos V.M., Saccenti E. (2021). In-pero: Exploiting deep learning embeddings of protein sequences to predict the localisation of peroxisomal proteins. Int J Mol Sci.

[17] Wanders R.J.A., Waterham H.R., Ferdinandusse S. (2016). Metabolic interplay between peroxisomes and other subcellular organelles including mitochondria and the endoplasmic reticulum. Front Cell Dev Biol.

[18] Islinger M., Voelkl A., Fahimi H., Schrader M. (2018). The peroxisome: an update on mysteries 2.0. Histochem Cell Biol.

[19] Islinger M., Grille S., Fahimi H.D., Schrader M. (2012). The peroxisome: an update on mysteries. Histochem Cell Biol.

[20] Brocard C., Hartig A. (2006). Peroxisome targeting signal 1: Is it really a simple tripeptide?, Biochimica et Biophysica Acta (BBA) - Molecular. Cell Res.

[21] Kamoshita M., Kumar R., Anteghini M., Kunze M., Islinger M., Martins dos Santos V., Schrader M. (2022). Insights into the peroxisomal protein inventory of zebrafish. Front Physiol.

[22] Cortes C., Vapnik V. (1995). Support-vector networks. Mach Learn.

[23] Baker A., Carrier D.J., Schaedler T., Waterham H., van Roermund C., Theodoulou F. (2015). Peroxisomal ABC transporters: functions and mechanism. Biochem Soc Trans.

[24] Aitchison J., Murray W.W., Rachubinski R. (1991). The carboxyl-terminal tripeptide ala-lys-ile is essential for targeting candida tropicalis trifunctional enzyme to yeast peroxisomes. J Biol Chem.

[25] De Hoop M., Ab G. (1992). Import of proteins into peroxisomes and other microbodies. Biochem J.

[26] Schlüter A., Real-Chicharro A., Gabaldón T., Sánchez-Jiménez F., Pujol A. (2009). Peroxisomedb 2.0: an integrative view of the global peroxisomal metabolome. Nucleic Acids Res.

[27] Alex Bateman, M.-J. Martin, S. Orchard, M. Magrane, R. Agivetova, S. Ahmad, E. Alpi, E.H. Bowler-Barnett, R. Britto, B. Bursteinas, H. Bye-A-Jee, R. Coetzee, A. Cukura, A.D. Silva, P. Denny, T. Dogan, T. Ebenezer, J. Fan, L.G. Castro, P. Garmiri, G. Georghiou, L. Gonzales, E. Hatton-Ellis, A. Hussein, A. Ignatchenko, G. Insana, R. Ishtiaq, P. Jokinen, V. Joshi, D. Jyothi, A. Lock, R. Lopez, A. Luciani, J. Luo, Y. Lussi, A. MacDougall, F. Madeira, M. Mahmoudy, M. Menchi, A. Mishra, K. Moulang, A. Nightingale, C.S. Oliveira, S. Pundir, G. Qi, S. Raj, D. Rice, M.R. Lopez, R. Saidi, J. Sampson, T. Sawford, E. Speretta, E. Turner, N. Tyagi, P. Vasudev, V. Volynkin, K. Warner, X. Watkins, R. Zaru, H. Zellner, A. Bridge, S. Poux, N. Redaschi, L. Aimo, G. Argoud-Puy, A. Auchincloss, K. Axelsen, P. Bansal, D. Baratin, M.-C. Blatter, J. Bolleman, E. Boutet, L. Breuza, C. Casals-Casas, E. de Castro, K.C. Echioukh, E. Coudert, B. Cuche, M. Doche, D. Dornevil, A. Estreicher, M.L. Famiglietti, M. Feuermann, E. Gasteiger, S. Gehant, V. Gerritsen, A. Gos, N. Gruaz-Gumowski, U. Hinz, C. Hulo, N. Hyka-Nouspikel, F. Jungo, G. Keller, A. Kerhornou, V. Lara, P.L. Mercier, D. Lieberherr, T. Lombardot, X. Martin, P. Masson, A. Morgat, T.B. Neto, S. Paesano, I. Pedruzzi, S. Pilbout, L. Pourcel, M. Pozzato, M. Pruess, C. Rivoire, C. Sigrist, K. Sonesson, A. Stutz, S. Sundaram, M. Tognolli, L. Verbregue, C.H. Wu, C.N. Arighi, L. Arminski, C. Chen, Y. Chen, J.S. Garavelli, H. Huang, K. Laiho, P. McGarvey, D.A. Natale, K. Ross, C.R. Vinayaka, Q. Wang, Y. Wang, L.-S. Yeh, J. Zhang, P. Ruch, D. Teodoro, Uniprot: the universal protein knowledgebase in 2021, Nucleic acids research 49 (D1) (2021) D480–D489. 10.1093/nar/gkaa1100 PMC7778908 33237286

[28] Cawley G.C., Talbot N.L.C. (2010). On over-fitting in model selection and subsequent selection bias in performance evaluation. J Mach Learn Res.

[29] Filzmoser P., Liebmann B., Varmuza K. (2009). Repeated double cross validation. J Chemometrics: J Chemometrics Soc.

[30] Platt J. (2000). Probabilistic outputs for support vector machines and comparisons to regularized likelihood methods. Adv Large Margin Classif.

[31] Refregier P., Vallet F. (1991). Artificial Neural Networks.

[32] Wu T.-F., Lin C.-J., Weng R.C. (2004). Probability estimates for multi-class classification by pairwise coupling. J Mach Learn Res.

[33] Savojardo C., Bruciaferri N., Tartari G., Martelli P.L., Casadio R. (2020). Deepmito: accurate prediction of protein sub-mitochondrial localization using convolutional neural networks. Bioinformatics.

[34] Wang X., Jin Y., Zhang Q. (2020). Deeppred-submito: a novel submitochondrial localization predictor based on multi-channel convolutional neural network and dataset balancing treatment. Int J Mol Sci.

[35] A. Morgat, T. Lombardot, E. Coudert, K. Axelsen, T.B. Neto, S. Gehant, P. Bansal, J. Bolleman, E. Gasteiger, E. de Castro, D. Baratin, M. Pozzato, I. Xenarios, S. Poux, N. Redaschi, A. Bridge, T.U. Consortium, Enzyme annotation in uniprotkb using rhea, Bioinformatics 36 (6) (2019) 1896–1901. 10.1093/bioinformatics/btz817 PMC7162351 31688925

[36] Li W., Godzik A. (2006). Cd-hit: a fast program for clustering and comparing large sets of protein or nucleotide sequences. Bioinformatics.

[37] Forcier J., Bissex P., Chun W.J. (2008).

[38] Matthews B.W. (1975). Comparison of the predicted and observed secondary structure of t4 phage lysozyme. Biochim Biophys Acta (BBA)-Protein Structure.

[39] Kohlwein S.D., Veenhuis M., van der Klei I.J. (2013). Lipid droplets and peroxisomes: Key players in cellular lipid homeostasis or a matter of fat-store ’em up or burn ’em down. Genetics.

[40] Sibirny A.A. (2016). Yeast peroxisomes: structure, functions and biotechnological opportunities. FEMS Yeast Res.

